# Supercritical CO_2_ Extraction as a Tool to Isolate Anti-Inflammatory Sesquiterpene Lactones from *Cichorium intybus* L. Roots

**DOI:** 10.3390/molecules26092583

**Published:** 2021-04-28

**Authors:** João P. Baixinho, José D. Anastácio, Viktoriya Ivasiv, Katarina Cankar, Dirk Bosch, Regina Menezes, Matthew de Roode, Cláudia Nunes dos Santos, Ana A. Matias, Naiara Fernández

**Affiliations:** 1 iBET, Instituto de Biologia Experimental e Tecnológica, Apartado 12, 2781-901 Oeiras, Portugal; jbaixinho@ibet.pt (J.P.B.); jose.diogo.anastacio@nms.unl.pt (J.D.A.); viktoriya.ivasiv@ibet.pt (V.I.); regina.menezes@nms.unl.pt (R.M.); claudia.nunes.santos@nms.unl.pt (C.N.d.S.); amatias@ibet.pt (A.A.M.); 2 CEDOC, Chronic Diseases Research Centre, NOVA Medical School|Faculdade de Ciências Médicas, Universidade NOVA de Lisboa, Campo dos Mártires da Pátria, 130, 1169-056 Lisboa, Portugal; 3 Wageningen University and Research, Wageningen Plant Research, BU Bioscience, Droevendaalsesteeg 1, 6708 PB Wageningen, The Netherlands; katarina.cankar@wur.nl (K.C.); dirk.bosch@wur.nl (D.B.); 4 Sensus B.V., Oostelijke Havendijk 15, 4704 RA Roosendaal, The Netherlands; matthew.de.roode@sensus.nl

**Keywords:** supercritical CO_2_ extraction, sesquiterpene lactones, anti-inflammatory potential

## Abstract

*Cichorium intybus* L. or chicory plants are a natural source of health-promoting compounds in the form of supplements such as inulin, as well as other bioactive compounds such as sesquiterpene lactones (SLs). After inulin extraction, chicory roots are considered waste, with most SLs not being harnessed. We developed and optimized a new strategy for SL extraction that can contribute to the conversion of chicory root waste into valuable products to be used in human health-promoting applications. In our work, rich fractions of SLs were recovered from chicory roots using supercritical CO_2_. A response surface methodology was used to optimize the process parameters (pressure, temperature, flow rate, and co-solvent percentage) for the extraction performance. The best operating conditions were achieved at 350 bar, 40 °C, and 10% EtOH as a co-solvent in a 15 g/min flow rate for 120 min. The extraction with supercritical CO_2_ revealed to be more selective for the SLs than the conventional solid–liquid extraction with ethyl acetate. In our work, 1.68% mass and a 0.09% sesquiterpenes yield extraction were obtained, including the recovery of two sesquiterpene lactones (8-deoxylactucin and 11β,13-dihydro-8-deoxylactucin), which, to the best of our knowledge, are not commercially available. A mixture of the abovementioned compounds were tested at different concentrations for their toxic profile and anti-inflammatory potential towards a human calcineurin/NFAT orthologue pathway in a yeast model, the calcineurin/Crz1 pathway. The SFE extract obtained, rich in SLs, yielded results of inhibition of 61.74 ± 6.87% with 50 µg/mL, and the purified fraction containing 8-deoxylactucin and 11β,13-dihydro-8-deoxylactucin inhibited the activation of the reporter gene up to 53.38 ± 3.9% at 10 µg/mL. The potential activity of the purified fraction was also validated by the ability to inhibit Crz1 nuclear translocation and accumulation. These results reveal a possible exploitable green technology to recover potential anti-inflammatory compounds from chicory roots waste after inulin extraction.

## 1. Introduction

At present, there is a growing interest in the potential benefits of nutritional compounds for disease prevention, which in turn also means an increasing demand for natural and nutritional food [1]. Biologically active compounds and functional ingredients (vitamins, antimicrobials, antioxidants, colorants, etc.) from natural sources are of great commercial interest, especially in the form of dietary supplements. The taproot of chicory (*Cichorium intybus* L., Asteraceae family) contains several health-related compounds such as inulin, flavonoids, vitamins, and sesquiterpene lactones (SLs) [2,3,4]. Chicory leaves have been consumed in Europe for many centuries, and the roots are currently used for the production of inulin, a fructose-based polymer that is added to numerous food products as a dietary fiber and sweetener [5]. After inulin extraction, the remaining biomass is considered waste, even though the roots are rich in other health-promoting compounds such as SLs. Several bioactivities have been previously associated with SLs, for instance, those that are antimicrobial, anti-inflammatory, and antiparasitic [3,5,6,7,8,9,10,11,12]. The most relevant SLs previously identified in chicory roots [13,14,15] include lactucin, lactucopicrin, 11β-13-dihydrolactucin, 11β-13-dihydrolactucopicrin, and 8-deoxylactucin [16].

SL extraction from several Asteraceae species has been carried out using different organic solvents or mixtures such as methanol, ethyl acetate, chloroform, n-hexane, and ethanol (EtOH) [17,18,19,20,21,22]. The need to minimize toxic solvent utilization has motivated the development of new environment-friendly processing technologies that can be carried out at the industrial scale. In this field, high pressure technologies are revealed to be remarkably efficient in enabling a more selective and effective recovery of high-value compounds from natural sources [23,24,25]. One such technology, supercritical fluid extraction (SFE), is generally performed using carbon dioxide (CO_2_). CO_2_ has a moderately low critical pressure and temperature (74 bar, 32 °C) and is nontoxic; it is available in high purity at relatively low costs and can be easily removed from the extract [26,27,28]. Supercritical CO_2_ is an excellent solvent for the extraction of nonpolar and low molecular weight compounds [25,29]. Solvent extraction power and selectivity have been widely studied and depend especially on SFE operating conditions, which includes the possibility of adding a co-solvent (usually water or EtOH) to modify supercritical CO_2_ polarity, enhancing target molecules solubility [23,25,26]. The combination of CO_2_ properties turns SFE into a sustainable and safe extraction technique for the extractions of ingredients for supplements and health care products [24,26,27].

The purpose of this study is to contribute to the valorization of chicory root by developing for the first time a high-pressure strategy to extract SLs for human health-promoting applications. *Chicorium intybus* L. SFE extract is highly rich in different SLs such as lactucin, lactucopicrin, 11β-13-dihydrolactucin, 11β-13-dihydrolactucopicrin, 8-deoxylactucin, and 11β,13-dihydro-8-deoxylactucin. After a chromatographic process, a purified fraction of a mixture of 8-deoxylactucin and 11β,13-dihydro-8-deoxylactucin could be isolated. The SFE extract and the purified fraction are tested in vitro for its toxicity at different concentrations as well its anti-inflammatory potential. In our study, the SFE extract was able to inhibit 61.74 ± 6.87% of the transcription factor activity with a concentration of 50 µg/mL an inhibition rate in the same range of those observed for the immunosuppressant pharmaceutical drug FK506 also known as tacrolimus. Furthermore, the purified fraction was also tested for the potential anti-inflammatory capacity, revealing a capacity to inhibit Crz1 activation by 53.38 ± 3.9% at 10 µg/mL.

## 2. Results and Discussion

### 2.1. Supercritical CO_2_ Extraction of SLs

Freeze-dried and milled (particle size < 1 mm) *Cichorium intybus* L. roots were subjected to SFE. Based on the preliminary extractions (data not shown) with supercritical CO_2_ as a solvent, the procedure resulted in a low mass yield extraction (0.25 mg extract/g raw material) while the mass yield increased when an organic co-solvent (10% EtOH) was used (10 mg extract/g raw material). An extraction kinetic was made (data not shown) at 300 bar and at 50 °C with a flow rate of 10 g/min, including 10% EtOH as a co-solvent, and 120 min was considered the best extraction time for the SFE processing of chicory roots.

**Figure 1:**
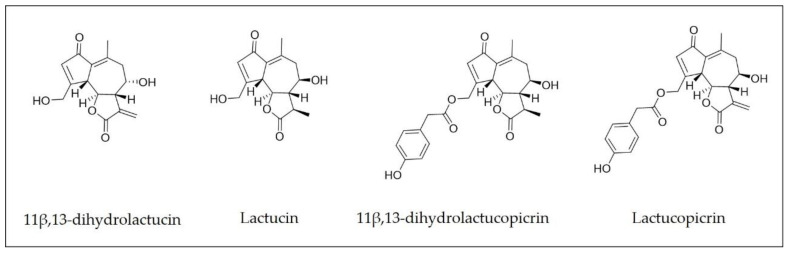
Major sesquiterpene lactones identified in *Cichorium intybus* L. root extracts.

A design of experiments (DOE) was planned in order to optimize the extraction procedure and to study the effect of process variables on the mass yield and SL concentration in the extract. SFE extracts were characterized by HPLC-DAD and were based on the SLs that are most abundant in chicory roots [8,14,18,30]. Four different compounds were identified by comparing the retention times of the commercially available SL standards: 11β,13-dihydrolactucin, lactucin, 11β,13-dihydrolactucopicrin, and lactucopicrin. The SL concentration in the extract was calculated based on the sum of the abovementioned SLs (Figure 1). In a first approach flow rate (10 g/min), the extraction time (120 min), and the co-solvent percentage (10% EtOH) were kept constant to evaluate the effect of temperature (40–80 °C) and pressure (100–550 bar) (see Table 1, DOE 1, two factors with three level face-centered composite design).

**Figure 2:**
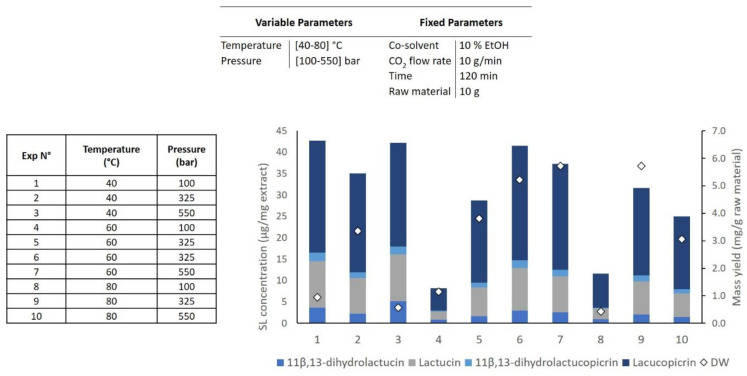
The extraction of SL was carried out following a Central Composite Face-Centered Design (DOE #1) as a function of pressure and temperature. Experiment numbers (1–10) correspond to the extraction number assay, white diamonds (values linked to right axis) represent extraction mass yield and coloured bars (values linked to left axis) represent the concentration of the major SLs extracted from *Cichorium intybus* L. roots from supercritical CO_2_ extraction process.

The obtained results regarding mass and SL concentration in the extract are shown in Figure 2. The regression analysis of the data shows that for a 90% confidence level (*p* < 0.10), the SL concentration in the extract was negatively affected by extraction temperature (*p* = 0.060). While the effect of CO_2_ pressure was not significant for the SL concentration, this parameter had a significantly quadratic effect on mass yield (*p* = 0.040), which means that the optimal levels of pressure are found in the middle of the experimental range.

As graphically described in Figure 2, the maximum mass yield was obtained (5.73 mg/g raw material) when the conditions depicted in assay 9 were applied (80 °C, 325 bar, and a continuous flow rate of 10 g/min CO_2_ with 10% of EtOH as the co-solvent), corroborating with the predicted results by the model. On the other hand, the minimum yield was achieved when the extraction was conducted at lower pressures (100 bar) as represented in assays 1, 4, and 8, with the lowest value (0.43 mg/g raw material) achieved at high temperatures (assay 8). Regarding the SL concentration in the extract, the effect of the temperature is visible when comparing extraction assays 1, 4, and 8. At the same pressure (100 bar), the SFE performed at a lower temperature (40 °C) resulted in an extraction four times higher in SLs (42.7; 8.2 and 11.6 µg/mg extract, respectively) compared to the ones performed at high temperature (80 °C). The most selective extraction is represented in assay 1 (42.7 µg/mg extract) and the lowest in assay 4 (8.2 µg/mg extract). Since no optimal extraction point was obtained with DOE 1, a second DOE (Table 1, DOE 2) was created to narrow the optimal extraction region based on previously obtained results with a fixed temperature at 40 °C and pressure at 350 bar. A set of experiments, including three center points, was carried out to optimize new variables, namely the co-solvent percentage (10–40% EtOH), flow rate (10–30 g/min), and low temperature (25–40 °C) in the extraction process.

**Figure 3:**
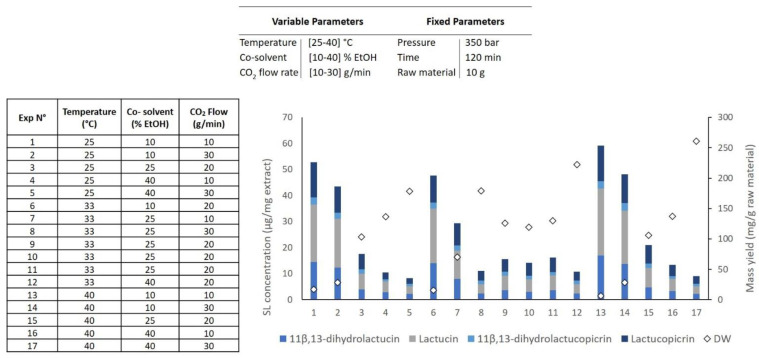
The extraction of SL was carried out following a Central Composite Face-Centered Design (DOE #2) as a function of pressure, temperature, flow rate and co-solvent percentage. Experiment numbers (1–17) correspond to the extraction number assay, white diamonds (values linked to right axis) represent extraction mass yield and coloured bars (values linked to left axis) represent the concentration of the major SLs extracted from *Cichorium intybus* L. roots from supercritical CO_2_ extraction process.

Figure 3 shows mass and SL concentration in the extract obtained in DOE 2. They were used to estimate the linear and quadratic effects of the variables and their synergies. The response surfaces (Figure 3) fitted to the SL concentration in the extract can be described by second-order polynomial models as a function of co-solvent percentage, temperature, and flow rate (Table 2). In these response surface models, for a 95% confidence level, the significant effects of *p* < 0.05 were included in the model equations. The high values for both R^2^ and R_adj_^2^ of these models (Table 2) suggest a close agreement between the experimental data and the theoretical values predicted by the model.

**Figure 4:**
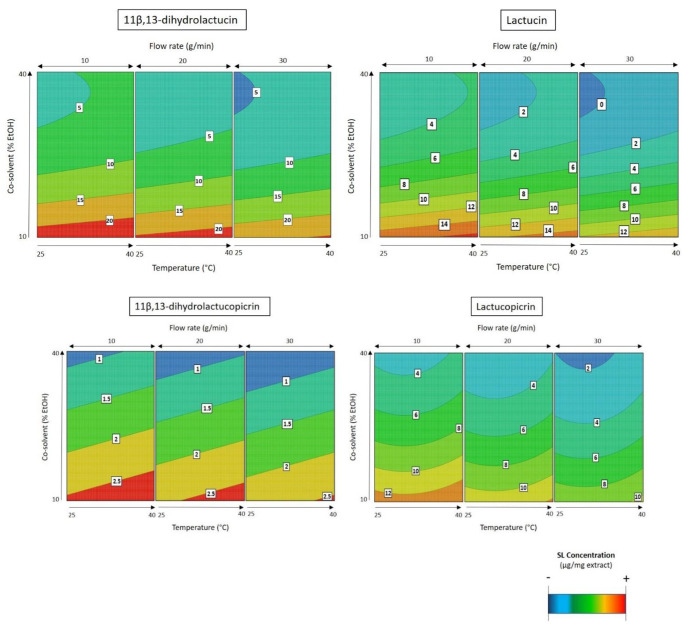
Response surfaces (DOE 2) fitted to the SL concentration in the extract as a function of temperature, co-solvent percentage (% EtOH), and flow rate. The white squares show the concentration (µg/mg extract) of each compound depending on the conditions of the three axes. In blue, the minimum extraction; in red, the maximum extraction achieved for each SL. To maximize SL extraction, the best SFE is conducted at high temperature (40 °C) with a low flow rate (10 g/min) and low co-solvent percentage (10% EtOH).

The analysis of the second response surface model for SL extraction (Figure 4) shows that there is a positive effect of temperature and a negative effect of co-solvent percentage and flow rate in the SL concentration in the extract. 

As already observed in the previous design of experiments, temperature is the most important parameter for the selectivity of the process. By varying the temperature again, it was possible to obtain the value that best favors the extraction of the SLs from *Cichorium intybus* L. roots. This positive effect means that the increase in temperature favors the extraction up to 40 °C, and beyond this value, this parameter has a negative effect in the process. As shown in Figure 4, the best temperature to extract all the four SLs is 40 °C.

When comparing all extraction attempts (Figure 3), we can see that the best selectivity results are obtained with low co-solvent percentages (assays 1, 2, 6, 13, and 14). Therefore, the best selectivity result was obtained when the extraction was conducted at 40 °C with a low flow rate (10 g/min) and low co-solvent percentage (10% EtOH). This extraction is characterized by presenting a 5.91% SL content on the final extract, and it is represented in assay 13 (Figure 3).

**Figure 5:**
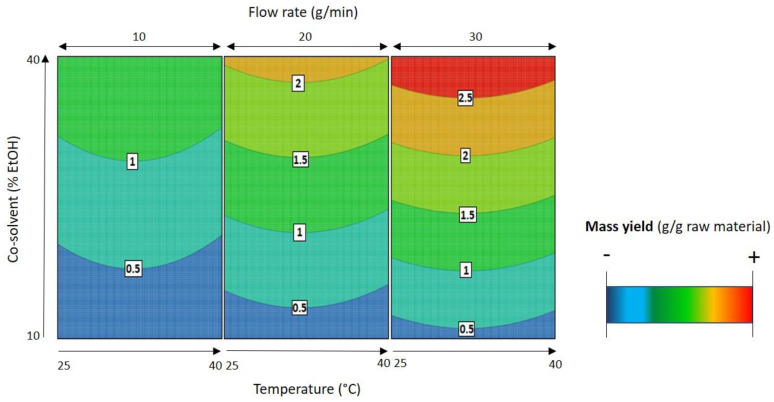
Response surfaces (DOE 2) fitted to the extraction mass yield (g/g raw material) as a function of temperature, co-solvent percentage (%EtOH), and flow rate. The white squares show the mass (mg) extracted depending on the conditions of the three axes. In blue, the minimum extraction; in red, the maximum mass extracted in SFE. To maximize mass yield, the best SFE is conducted at a high flow rate (30 g/min) with a high co-solvent percentage (40% EtOH).

In contrast, the mass yield was higher when the extraction process was performed at a high flow rate (30 g/min) with high co-solvent percentages (40% EtOH) (assays 5, 12, and 17; Figure 3). This result was expected because a reduced selective extraction is obtained when high flow rates and co-solvents are applied in SFE. However, an increase in extract mass does not mean an increase in SL content, as shown in Figure 3. As it can be noted, when the polarity of the extractant solvent is changed, the final mass characterization can also be altered. Moreover, as the solvent (CO_2_ + EtOH) flow rate was increased, independently from the temperature applied, the extraction capacity of the described system increased (red area, Figure 5). Here, the best conditions to increase the mass yield were 40 °C, 30 g/min flow rate with 40% EtOH as the co-solvent, resulting in a 260 mg/g raw material with 0.91% of SLs in its composition (assay 17; Figure 3).

An optimal condition to maximize both responses (mass yield and SL yield) was not obtained, but a region where all criteria were met was found at 350 bar, 40 °C with a continuous flow rate of 13–19 g/min flow with 10% EtOH as a co-solvent; this region is commonly called the sweet spot.

From the sweet spot, the best operating conditions were set, corresponding to a 120 min, at 350 bar, 40 °C, and 15 g/min flow with 10% EtOH as the co-solvent. This optimization resulted in a 16.8 mg/g raw material extraction with 53 µg/mg extract of SLs in its composition, accounting for an acceptable balance between mass yield and SL concentration in the extract. 

### 2.2. Conventional Solid–Liquid Extraction of SL 

SFE performance was compared to the conventional solvent extraction. Water and ethyl acetate were used as two different solvents to extract SLs from freeze-dried and milled *Cichorium intybus* L. roots. Overall, the two extractions resulted in a low SL concentration, i.e., 7.5 and 6.6 µg/mg extract, respectively. Although the mass yield was higher in the water extract (31%) compared to SFE extraction (1.68%), the ethyl acetate extract resulted in a similar mass yield (1.54%). This fact is explained by the solvent extraction power as the capacity of water to extract compounds with a variety of polarities [4,31,32]. 

### 2.3. Fractionation of Cichorium intybus L. Extract

**Figure 6:**
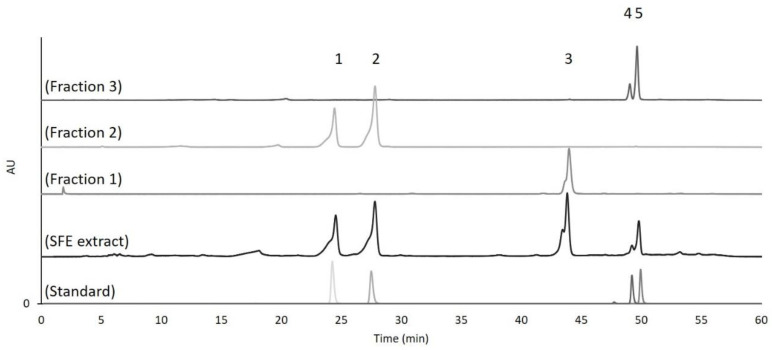
HPLC-DAD chromatograms (λ = 480 nm) of standards (numbers represent the different SL obtained in the corresponding fraction: 1-11β,13-dihydrolactucin; 2-Lactucin; 3-8-deoxylactucin and 11β,13-dihydro-8-deoxylactucin; 4-11β,13-dihydrolactucopicrin; 5-Lactucopicrin), the best SFE extract (350 bar, 40 °C, 10% EtOH as co-solvent and 15 g/min flow rate during 120 min) and the three different fractions obtained by flash chromatography using Silica gel 60 mesh as stationary phase and Dichloromethane:Methanol (95:5 *v*/*v*) as mobile phase.

The best SFE chicory extract was fractionated by flash column chromatography. Three different fractions were obtained: fraction 1 (retention factor = 0.79; 6.99% purification yield), fraction 2 (retention factor = 0.62; 7.01% purification yield), and fraction 3 (retention factor = 0.53, 2.82% purification yield) The fractions were analyzed by HPLC and 11β,13-dihydrolactucin and lactucin were identified in fraction 2 and 11β,13-dihydrolactucopicrin and lactucopicrin in fraction 3 (Figure 6). 

**Figure 7:**
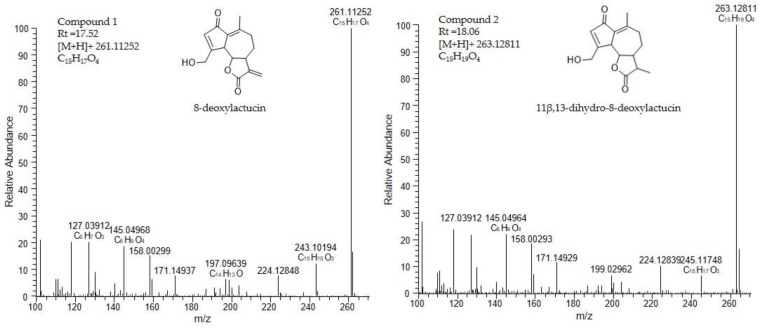
Mass spectrum of the compounds 1 and 2. Compound 1 was identified as 8-deoxylactucin, and compound 2 was identified as 11β,13-dihydro-8-deoxylactucin.

Fraction 1 could not be identified with the available SL standards, and so it was then analyzed using LC-MS (Figure 7). The analysis showed that two compounds were present in that fraction. Compound 1 eluting at the retention time of 17.5 min, with the accurate mass [M+H]^+^ of 261.11252, corresponds to the elemental formula C_15_H_16_O_4_ typical of 8-deoxylactucin (at 1.47 ppm mass accuracy). Compound 2 eluting at the retention time of 18.06 min, with accurate mass [M+H]^+^ of 263.12811, corresponds to the elemental formula C_15_H_18_O_4_, characteristic of 11β,13-dihydro-8-deoxylactucin (at 1.23 ppm mass accuracy). Both compounds were previously described in chicory extracts [17,33].

### 2.4. SFE Extract Potential Anti-Inflammatory Activity 

**Figure 8:**
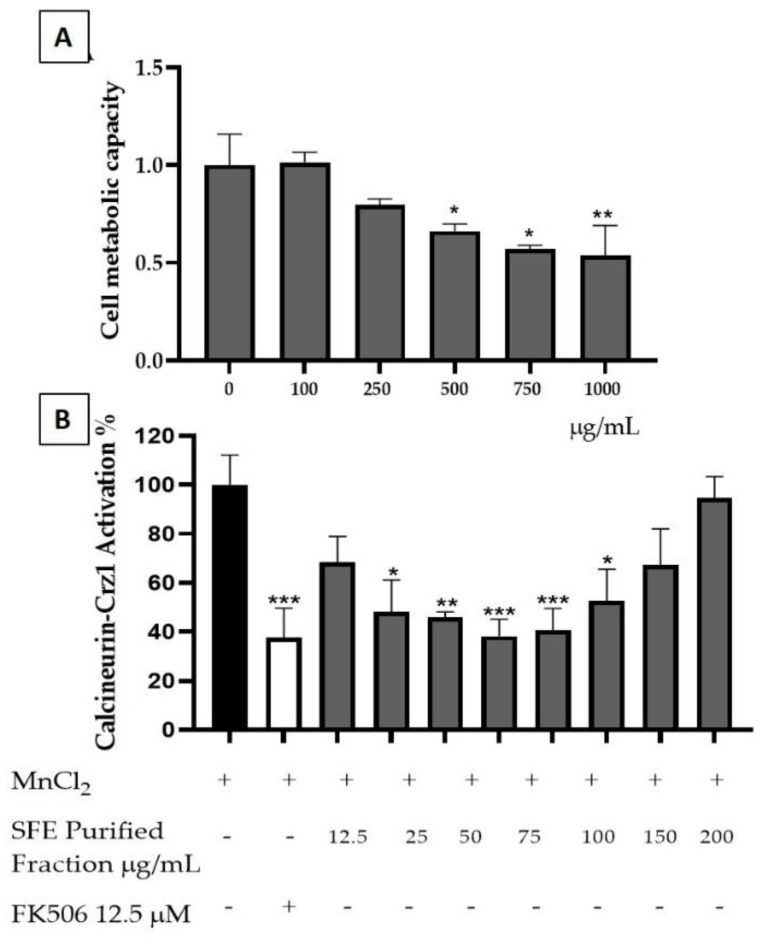
SFE extract potential for inhibiting calcineurin/Crz1 activation. (**A**) Toxicity of SFE extract was evaluated at the indicated concentrations of SFE in µg/mL. (**B**) Anti-inflammatory potential of SFE extract as demonstrated by the inhibition of the calcineurin/Crz1 pathway in *S. cerevisiae*. Statistical differences are noted as * *p* < 0.1, ** *p* < 0.01, *** *p* < 0.001 relative to the activated control (cells treated with MnCl_2_).

The SFE extracts were then tested for their potential for anti-inflammatory activity. Cytotoxicity evaluation on yeast cells showed a toxicity for concentrations higher than 500 µg/mL (Figure 8A). The potential of an SFE extract to attenuate inflammatory responses through the modulation of Crz1, the yeast orthologue of the human nuclear factor of activated T-cells (NFAT) pathway, was also evaluated for the nontoxic concentrations of the SFE extract (Figure 8B). SFE demonstrated a higher anti-inflammatory potential at concentrations such as 50 and 75 µg/mL, reducing the calcineurin/Crz1 pathway activation, up to 61.74 ± 6.87% and 59.5 ± 0.04%, respectively, at rates statistically comparable to the positive control FK506 (62.48 ± 2.10%) with a calculated IC_50_ of 38.81 ± 0.32 µg/mL (Figure 8B). Both in human and in yeast, i.e., NFAT and Crz1, respectively, are under the control of calcineurin. Human calcineurin and yeast calcineurin share a high evolutionary similarity, making this model ideal for the screening of compounds with anti-inflammatory potential. In the presence of a stimulus (MnCl_2_), Crz1 dephosphorylates from calcineurin and migrates towards the nucleus, thus promoting the expression of calcineurin dependent response element (CDRE) regulated genes similarly to human NFAT. SFE extract is rich in SLs, which have been already demonstrated to possess anti-inflammatory potential in a previous study [34]. However, this extract contains unexplored SLs such as the 8-deoxylactucin and 11β,13-dihydro-8-deoxylactuin, whose anti-inflammatory activity was not previously described towards the calcineurin/Crz1 pathway. 

### 2.5. Purified Fraction Anti-Inflammatory Potential

**Figure 9:**
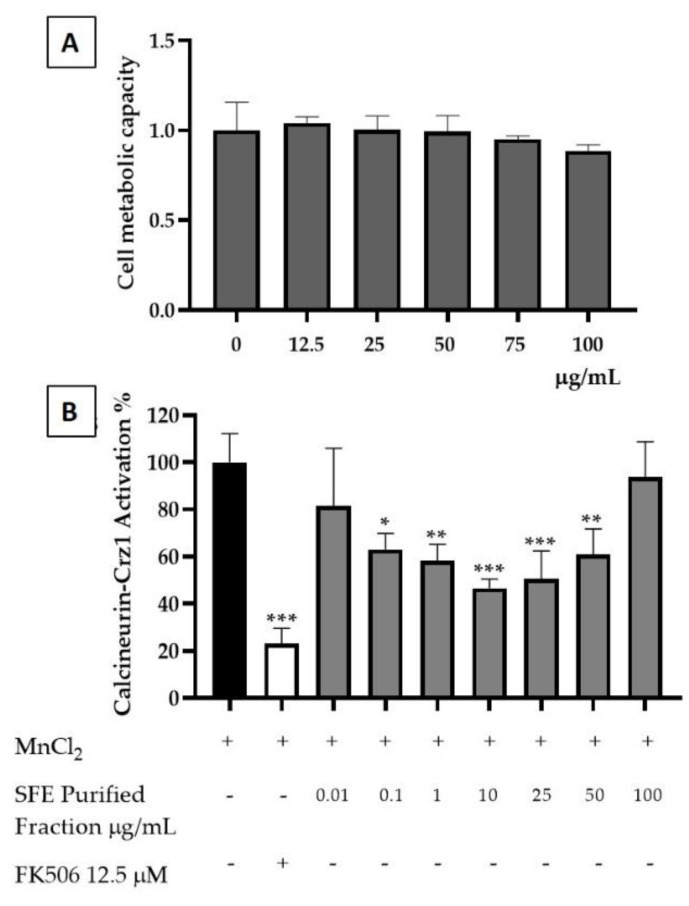
Purified fraction potential for inhibiting calcineurin/Crz1 activation. (**A**) Toxicity was evaluated at the indicated concentrations of the SFE purified fraction. (**B**) Anti-inflammatory potential of SFE purified fraction demonstrated by the inhibition of the calcineurin/Crz1 pathway in *S. cerevisiae*. Statistical differences are noted as * *p* < 0.1, ** *p* < 0.01, *** *p* < 0.001 relative to the activated control (cells treated with MnCl_2_).

As mentioned above, other pure SLs present in the SFE extract, such as lactucin, lactucopicrin, 11β,13-dihydrolactucin and 11β,13-dihydrolactucopicrin, all of which are commercially available, were already investigated for their anti-inflammatory potential in our previous study [34]. Therefore, we decided to explore the potential of this novel purified fraction containing a mixture of 8-deoxylactucin and 11β,13-dihydro-8-deoxylactucin. In order to measure its associated toxicity, yeast cells were exposed to different concentrations of SFE purified fraction. Here, 8-deoxylactucin and 11β,13-dihydro-8-deoxylactucin demonstrated no toxicity towards yeast cells at concentrations ranging from 12.5 µg/mL to 100 µg/mL (Figure 9A).

The SFE purified fraction’s ability to reduce the activation of calcineurin/Crz1 was tested as an indicator of an anti-inflammatory potential. The fraction presents its maximum activity at 10 µg/mL, promoting a reduction in Crz1 activation of 53.38% ± 3.9 (Figure 9B). This result demonstrated that this compound is able to reduce the activation of the calcineurin/Crz1 pathway at levels similar to those of the immunosuppressant drug used as a positive control FK506 with a calculated IC_50_ of 7.2 ± 3.15 µg/mL. 

The SL 8-deoxylactucin contains an α-methylene-γ-lactone ring in its structure. This moiety was described to be the most bioactive group present in SLs with respect to anti-inflammatory activity by interacting with proteins that contain exposed sulfhydryl groups via Michael-type addition [35]. This compound may be able to interact with calcineurin or through its exposed sulfhydryl groups present in its catalytic subunit [36,37]. Although α-methylene-γ-lactones rings are the most bioactive group, they may also present the ability to create conjugates via Michael-type addition with glutathione intracellularly—a process already described for other chicory SLs, lactucin and lactucopicrinand other SLs such as noblin—that become unavailable for further reaction with the target proteins [38,39].

In a previous study, chicory SL 11β,13-dihydrolactucin, lacking the α-methylene-γ-lactone ring, was able to modulate the activation of calcineurin/Crz1 pathway due to the presence of α,β-unsaturated carbonyl groups [34]. In addition, 11β,13-dihydro-8-deoxylactucin also does not contain the α-methylene-γ-lactones group, but it contains other functional groups such as α,β-unsaturated carbonyl, similarly to 11β,13-dihydrolactucin, possibly still being able to modulate the calcineurin/Crz1 pathway acting as a potential anti-inflammatory compound [34].

**Figure 10:**
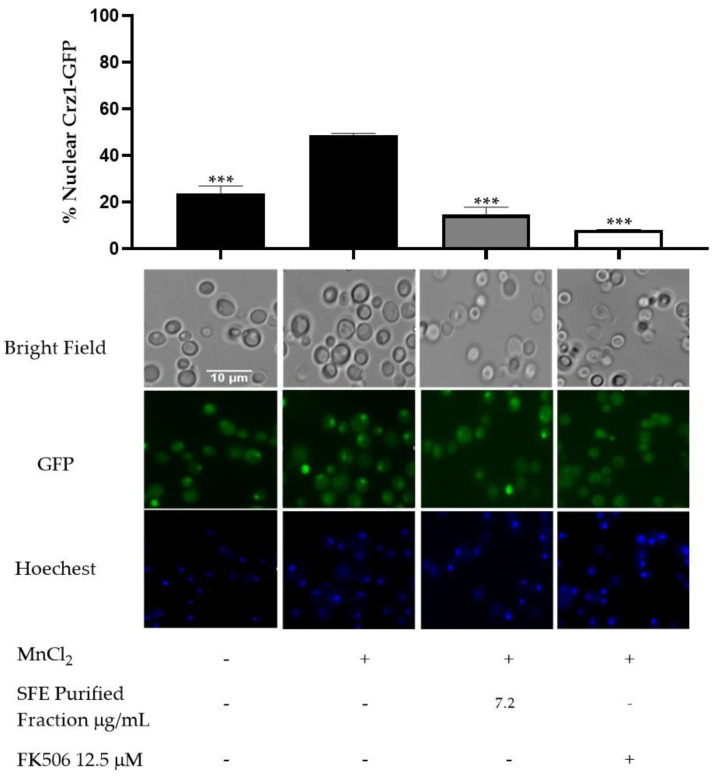
Purified fraction inhibits Crz1–GFP nuclear accumulation. Yeast cells were first treated either with or without 7.2 µg/mL of purified fraction, challenged with 3 mM MnCl_2_, and Crz1–GFP subcellular distribution was monitored using fluorescence microscopy. The immunosuppressant FK506 was used as a positive control. Approximately 1800 cells representing each condition were counted. Representative imagens are shown, and the values are the mean of percentage of nuclear Crz1–GFP ± SD of three biological replicates. Statistical differences are denoted as *** *p* < 0.001 relative to activated cells.

To confirm the effect of purified fraction containing 8-deoxylactucin and its dihydro variant on Crz1 activity, fluorescence microscopy was used as a means to assess their interference with Crz1 nuclear translocation and the accumulation of the transcription factor Crz1 at the concentration of the previously calculated IC_50_ of the fraction (7.2 µg/mL). The Crz1 signal is dispersed throughout the cells in the control condition, challenging cells with MnCl_2_ triggered Crz1 nuclear accumulation (48.7%), which was inhibited by SFE purified fraction treatment (14.6%) in a similar manner to that of the positive control FK506, known to inhibit calcineurin activity and thereby preventing the translocation of Crz1 to the nucleus (Figure 10).

## 3. Materials and Methods

### 3.1. Chemicals

CO_2_ pure grade (99.95%, Air Liquide, Lisbon, Portugal) and EtOH (96%) were used for high pressure extraction experiments. Solvents used for conventional extractions purification steps and chromatographic analysis included ethyl acetate (99.98%, Fisher scientific U.K. Limited, Loughborough, UK), methanol (99.8%, Fisher scientific U.K. Limited, Loughborough, UK), dichloromethane (Honeywell Riedel-de Haën, Germany), distilled water, and ultrapure water purified with a Milli-Q water purification system (Merck Millipore, Billerica, MA, USA). For the inflammation assays, the inflammatory stimulus was carried out by the usage of MnCl_2_ (Merck, Darmstadt, Germany), and the anti-inflammatory positive control was carried by FK506 (Cayman Chemicals, Ann Arbor, MI, USA). Cellular lysis was preformed using Yeast Protein Extraction Reagent (Y-PER; ThermoFisher Scientific, Scientific, Rockford, IL, USA). To quantify the expression of reporter gene lacZ, we employed a solution buffer composed of Na_2_HPO_4_ (ROTH, Karlsruhe, Germany), NaH_2_PO_4_·H_2_O (Merck, Buchs, Switzerland), KCl (Panreac, Barcelona, Spain), and MgSO_4_·7H_2_O (Merck, Buchs, Switzerland), containing o-nitrophenyl β-D-galactopyranoside (ONPG; Sigma–Aldrich^®^–Poole, Dorset, UK). Nuclear staining was carried out using nuclear dye Hoechst 33342 (Sigma, Buchs, Switzerland).

### 3.2. Raw Material

Chicory roots were obtained from a processing industry (Sensus, The Netherlands), which were harvested in November 2018. For sample preparation, roots were received, cleaned, and cut into pieces (1 cm) for freeze-drying (Coolsafe Superior Touch 55-80, Scanvac). The samples were further milled, and the particle size was determined using an AS 200 basic vertical vibratory sieve shaker (Retsch, Haan, Germany), with a measuring range between 250 and 1000 µm. The milled roots were stored and protected from light while kept at room temperature in a desiccator until the day of the experiments.

### 3.3. Extraction and Fractionation Procedures

#### 3.3.1. Supercritical CO_2_ Extractions

High-pressure extractions were carried out in an SFE system (Thar Technology, Pittsburgh, PA, USA, model SFE-500F-2-C50) [40]. Liquid CO_2_ was fed into the extraction vessel using a Thar SFC P-50 high pressure pump (Thar Technology, Pittsburgh, PA, USA). The extraction vessel was filled with 10 g of freeze-dried, milled chicory roots and glass beads in order to decrease solvent consumption. EtOH used as a co-solvent was pumped to the extraction vessel using a Thar SFC P-50 high pressure pump (Thar Technology, Pittsburgh, PA, USA). The vessel was pressurized with CO_2_ until reaching the required pressure (100–550 bar), which was maintained by an automated back pressure regulator (Thar SFC ABPR, Thar Technology, Pittsburgh, PA, USA). CO_2_ was expanded into the first fraction collector, and the extracts were recovered in a flask placed in an ice bath.

#### 3.3.2. Experimental Design Analysis/Statistical Analysis

Response surface methodology, a collection of statistical and mathematical techniques for improvement and optimize processes, is proposed to model the effect of different parameters such as pressure, temperature, co-solvent percentage, and flow rate on the extraction performance by SFE [41,42,43].

In this study, response surface methodology was used to model the recovery of SLs from chicory roots and to optimize the SFE process conditions. The extraction of SL was carried out following two different central composite face-centered (CCFC) designs as a function of pressure (100–550 bar), temperature (25–80 °C), flow rate (10–30 g/min), and co-solvent percentage (10–40%) to optimize the mass and SL extraction yield. After the selection of independent variables and their ranges, experiments were established based on a CCFC design, and each one was coded at three levels, i.e., −1, 0, and +1 (Table 1). The results of the CCFC, particularly the total mass and the SL concentration in the extract value, were analyzed using the software MODDE (MODDE Go 12.1 32-bit, Umetrics, Sweden). Both linear and quadratic effects of each factor under study, as well as their interactions, were calculated based on different extractions that were performed in a random order, as defined by the software. A surface, as described by a second-order polynomial equation, was fitted to each set of experimental data points. First- and second-order coefficients of the polynomial equations were generated by regression analysis. The fit of the models was evaluated using the determination coefficients (R^2^) and adjusted R^2^ (R_ad_j^2^).

#### 3.3.3. Conventional Solid–Liquid Extraction

The solid–liquid extraction, a process implemented by an industrial project partner (Sensus, The Netherlands), was performed for comparison with the SFE results. Briefly, the freeze-dried and milled *Cichorium intybus* L. roots were extracted with either ethyl acetate or water (raw material:solvent 1:10 (*w*/*v*)) for 1 h at 60 °C with mechanic stirring (RW20.n IKA, Labortechnik, Germany) at 900 rpm. Then, the extract was filtered (qualitative filter paper, 125 mm, from Filter-Lab) and centrifuged (Eppendorf Centrifuge 5810 R) to remove any remaining solid part of the unsolved raw material. The extract was dried in a rotary evaporator under reduced pressure at 40 °C. The experiments were performed in triplicate.

#### 3.3.4. Flash Column Chromatography Purification

Purification of the crude extract obtained from SFE consisted of dry loading approximately 1.5 g of the sample into a chromatographic column (30 × 400 mm) packed with silica gel 60 (0.04–0.06 mm, 230–400 mesh ASTM). The eluent for the purification was dichloromethane:methanol (9.5:0.5), which was used with an isocratic flow. Thin-layer chromatography (TLC) was conducted with TLC silica gel 60 F 254 and visualized under UV light to follow the purification, using the same eluent system. The pure fractions were collected, and the solvent was evaporated in a rotary evaporator under reduced pressure at 40 °C, for further analysis.

### 3.4. SL Analysis

#### 3.4.1. Quantification of SL by HPLC with Diode-Array Detector (HPLC-DAD)

HPLC analyses were carried out on a Thermo HPLC Dionex Ultimate 3000, equipped with a quaternary pump, solvent degasser, autosampler, and column oven, coupled to a Photodiode Array Detector Thermo Dionex DAD-3000. The separation was performed on a reversed-phase column (LiCrospher^®^ 100 RP-18, 250 × 4 mm; 5 mm; Merck^®^) at 35 °C. Elution was carried out in gradient mode employing the following solvent system: mobile phase A, methanol/water 14/86 (*v*/*v*); mobile phase B, methanol/water 64/36 (*v*/*v*). The gradient program used was 0 to 20 min, 100–58% A; 20 to 30 min, 58% A; 30 to 45 min, 58–0% A; 45 to 50 min, 0% A; 50 to 52 min, 0–100% A; 52 to 62 min, 100% A. The flow rate used was 0.5 mL/min, and the injection volume was 20 µL. A photodiode array detector was used to scan the wavelength absorption from 210 to 600 nm. The software used was Thermo Scientific™ Dionex™ Chromeleon™ (version 7.2 SR4) processed data.

#### 3.4.2. Identification of SL by Liquid Chromatography–Mass Spectrometry (LC-MS)

For LC-MS analysis purified fraction 1 was dissolved in 70% methanol containing formic acid (0.1%) at the concentration of 10 μg/mL. LC-MS analysis was performed using the LC-PDA-LTQ-Orbitrap FTMS system (Thermo Scientific), which consists of an Acquity UPLC (H-Class) with Acquity eLambda photodiode array detector (220–600 nm) connected to a LTQ/Orbitrap XL hybrid mass spectrometer equipped with an electrospray ionizator (ESI). The injection volume was 5 μL. Chromatographic separation was conducted on a reversed phase column Luna C18 column (2.0 × 150 mm, 3 µm; Phenomenex, USA) at 40 °C. Degassed eluent A (ultra-pure water: formic acid (1000:1, *v*/*v*)) and eluent B (acetonitrile:formic acid (1000:1, *v*/*v*)) were used at a flow rate of 0.19 mL min^−1^. A linear gradient from 5 to 75% acetonitrile (*v*/*v*) in 45 min was applied, which was followed by 15 min of washing and equilibration. FTMS full scans (*m*/*z* 90.00–1350.00) were recorded with a resolution of 60,000.

### 3.5. Anti-Inflammatory Evaluation

#### 3.5.1. Saccharomyces Cerevisiae Strains and Growth Conditions

The *S. cerevisiae* strains used in this study were YAA3 (his3::CRZ1-GFP-HIS3) and YAA5 (aur1::AUR1-C-4xCDRE-lacZ) [39]. The growth conditions were similar to those previously described [34]. Briefly, a preinoculum was prepared in an SC (synthetic complete) medium (0.79% (*w*/*v*) CSM (MP Biomedicals, Inc.—Fisher Scientific, Irvine, CA, USA), 0.67% (*w*/*v*) YNB (DifcoTM Thermo Scientific Inc., Waltham, MA, USA), and 2% (*w*/*v*) glucose), and cultures were incubated overnight at 30 °C under orbital shaking. Cultures were diluted in a fresh medium and incubated under the same conditions until the optical density at 600 nm (OD600) reached 0.5 ± 0.05. Readings were performed in a 96-well microtiter plate using a Biotek Power Wave XS plate spectrophotometer (Biotek^®^ Instruments, Winooski, VT, USA).

#### 3.5.2. Cell Viability Assays

Cell viability was monitored as previously described [34]. Briefly, 100 μL of cultures diluted to a final OD600 = 0.025 ± 0.0025 were placed into 96-well microplates; 10 μL of cell titer blue reagent (Promega, WI, USA) were added, and the plates were incubated for 3 h at 30 °C. Fluorescence was measured in 30 min intervals at emission wavelength 580 nm using the Biotek Power Wave XS Microplate Spectrophotometer (Biotek^®^ Instruments, Winooski, VT, USA). Doses of chicory SFE extracts ranging from 100 to 1000 µg/mL, and purified fractions containing 8-deoxylactucin and 11β,13-dihydro-8-deoxylactucin in the range of 12.5–100 µg/mL were tested in the viability assays. Readings were performed using a Biotek Power Wave XS plate spectrophotometer (Biotek^®^ Instruments, Winooski, VT, USA).

#### 3.5.3. Measurement of Reporter Gene Activity

Measurement of β-galactosidase activity was performed as previously described [34]. Briefly, cells were challenged with an SFE chicory extract with concentrations ranging from 1 µg/mL to the highest nontoxic concentration. For the purified fraction of the SFE extract containing 8-deoxylactucin and 11β,13-dihydro-8-deoxylactucin, the concentrations tested ranged from 10 ng/mL up to 100 µg/mL. The immunosuppressant drug FK506 (Cayman Chemicals, Ann Arbor, MI, USA) was used as a positive control at a final concentration of 12.5 µM. The final results were expressed as Miller units (MU) according to the following equation, where V is the volume of culture assayed in mL, and t is the reaction time in minutes [44]:

Miller unit = 1000 × (OD420 − 1.75 × OD550)/(t × V × OD600)

#### 3.5.4. Fluorescence Microscopy

The monitoring of Crz1 subcellular localization was performed as previously described [34]. Briefly, cells were treated with 7.2 µg/mL of purified fraction of SFE extract containing the SLs 8-deoxylactucin and its dihydro variant, or12.5 µM of FK506. The preparations were monitored for GFP fluorescence as previously described using a Zeiss Imager Z2 (Zeiss, Germany) fluorescence microscope [45]. Photographs were taken with an Axiocam 506 mono (Zeiss). At least 600 individual cells were counted for each condition. Images were analyzed using Fiji-ImageJ1.53c (United States).

### 3.6. Statistical Assays

Results for the toxicity and bioactivity of SFE extract and fraction are the averages of three independent experiments and are reported as mean ± SD. Differences between the controls and the experimental concentrations were assessed by an analysis of variance with Dunnett’s multiple comparison tests (α = 0.05), utilizing GraphPad Prims 8.4.2 software. The IC_50_ value for the SFE total extract and the SFE purified fraction was also calculated using the GraphPad Prims 8.4.2 software.

## 4. Conclusions

In this study, a green extraction approach using supercritical CO_2_ was explored for the first time to recover SLs from *Cichorium intybus* L. roots. The best operating conditions to obtain sesquiterpene-rich extracts were achieved at 350 bar and 40 °C in a 15 g/min flow rate (10% EtOH). SFE proved to be an efficient process for extracting SL (53 µg/mg extract) while reducing the quantity of organic solvents consumed. The SFE method produced an extract rich in SLs, compounds that had been previously described as anti-inflammatory in *S. cerevisiae* model. Moreover, a purified fraction containing a mixture of noncommercially available 8-deoxylactucin and 11β,13-dihydro-8-deoxylactucin was obtained. The SFE extract was then tested for its anti-inflammatory potential, where it was able to decrease the activity of the calcineurin/NFAT orthologue Crz1 in a *S. cerevisiae* model with an IC_50_ of 38.81 ± 8.32 µg/mL, indicating the possibility of exploiting this extraction method to produce a possible anti-inflammatory extract using green technology with reduced costs. The purified fraction’s anti-inflammatory capacity was also measured, resulting in an inhibition of the pathway with an IC_50_ of 7.2 ± 3.15 µg/mL, which are results similar to those of the immunosuppressant pharmaceutical drug FK506. We also verified the ability of this purified fraction to inhibit the translocation of the transcription factor Crz1 to the nucleus by cellular localization, observed by fluorescence microscopy. Calcineurin is highly conserved among eukaryotes, and the docking of the NFAT sequence recognized by calcineurin is highly similar to Crz1. This makes the yeast model suitable for studying the interaction of compounds with the mammalian equivalent calcineurin-NFAT pathway. However, further studies are required in order to validate this anti-inflammatory potential achieved through the inhibition of NFAT using other advanced mammalian cellular models and to address the molecular targets of the purified fraction. NFAT nuclear translocation and target genes expression can confirm the potential previously observed. A final validation in animal models subjected to an inflammatory insult is ultimately required for a deeper understanding of the mechanisms, doses, and role of the compounds on inflammatory biomarkers.

Based on these results, we can consider pressurized fluid extraction technology as a good alternative to traditional solvent extraction methods for the development of high-value products from *Cichorium intybus* L. with potential human health-promoting applications.

## Figures and Tables

**Table 1:** Variables used in the two different design of experiments (DOE) and actual values of variables for the coded values. First process optimization (DOE 1) with 2 process variables, i.e., temperature and pressure, and after the final process parameters optimization (DOE 2) with 3 process variables, i.e., flow rate, co-solvent percentage, and temperature.

		Levels		
	Variable, unit	−1	0	1
**DOE 1**	Temperature, °C	40	60	80
	Pressure, bar	100	325	550
**DOE 2**	Temperature, °C	25	33	40
	Co-solvent, % EtOH	10	25	40
	Flow rate, g/min	10	20	30

**Table 2:** Model equations for the response surfaces, after final SFE optimization (DOE 2), fitted to the values of mass yield (MY) and the four major SLs extracted as a function of temperature (A), co-solvent percentage (B), and flow rate (C), and their respective R^2^ and R_adj_^2^.

Polynomial Model Equations	R^2^	R_adj_^2^
MY = 1.232 + 0.936A + 0.403B − 0.124A2 + 0.285BC	0.99	0.98
11β,13-dihydrolactucin = 4.2 + 0.748A − 6.096B − 1.519C + 4.01B2	0.98	0.97
Lactucin = 6.262 + 1.046A − 9.261B − 2.155C + 6.088B2	0.98	0.98
11β,13-dihydrolactucopicrin = 1.688 − 0.876B	0.90	0.87
Lactucopicrin = 5.564 − 4.293B − 1.271C + 1.236B2	0.96	0.94

## Data Availability

Not applicable.

## Sample Availability

Samples of the compounds are available on request from the authors.
